# Amphioxus, motion detection, and the evolutionary origin of the vertebrate retinotectal map

**DOI:** 10.1186/s13227-018-0093-2

**Published:** 2018-02-19

**Authors:** Thurston Lacalli

**Affiliations:** Biology Department, University of Victoria, Victoria, BC V8W-3N5 Canada

**Keywords:** *Branchiostoma*, Chordate evolution, Vision, Retina, Optic nerve, Optic tectum

## Abstract

The axonal projection from the retina to the optic tectum maps visual information isomorphically from one to the other and serves as a model for the development of sensory maps more generally in the vertebrate brain. How or why this connection evolved is not known, nor why the midbrain is so important to the processing of visual information. Amphioxus is potentially informative here because its eye homolog, the frontal eye, also has a neural connection to a region of the brain now known to be homologous with the caudal diencephalon and midbrain. The frontal eye has only a one-dimensional receptor array, but simple alterations to the pattern and plane of cell division would have been sufficient to generate a structure more like the vertebrate retina. Accounting for the retinotectal map poses more of a problem. The hypothesis developed here is that this is best explained as a consequence of a prior association between the roof of the anterior nerve cord and an array of rhabdomeric photoreceptors, homologous with the Joseph cells of amphioxus, that were used by the common ancestor of amphioxus and vertebrates for detecting moving shadows. Hence, a rudimentary tectal map could have been present before the evolution of image-forming eyes and been coopted by them secondarily. Assuming the orientation of this map was fixed from the start relative to the external world, its retinal counterpart would have had to adjust to this to accommodate the image reversal that accompanies the conversion of a flat receptor array to a camera-type eye. Exploring this hypothesis further will require more information than is currently available on the Joseph cells, especially as to where and how their neural output is processed.

## Background

The vertebrate retina maps to the optic tectum (the superior colliculus in mammals) in an isomorphic fashion, so the visual field as registered by the former is essentially reproduced on the surface of the latter. This provides an experimentally accessible model for investigating the developmental and molecular mechanisms that generate well-ordered sensory maps and has contributed to our understanding of the development of brain circuits more generally [1, 2]. How and why the retinotectal projection originated in evolution is less clear, largely because there are no intermediate stages to bridge the evolutionary gap between the simple photoreceptors of basal deuterostomes, including invertebrate chordates, and the visual system of vertebrates. Amphioxus is currently considered the best available model for ancestral chordates [3], but features only peripherally in most accounts of eye evolution (for an exception, see Fig. 5.5 in [4]). Instead, in the absence of a generally accepted precursor for the vertebrate eye among non-vertebrate taxa, the structurally simple eyes of hagfish and larval lampreys have typically served as proxies for the ancestral eye [5, 6]. The situation has now changed with the recognition, based on electron microscopical (TEM) and molecular data [7–10], that the vertebrate eye and the frontal eye of amphioxus are almost certainly homologs. It is therefore timely to consider whether this alters our understanding of early events in the evolution of the vertebrate visual system in significant ways.

**Fig. 1:**
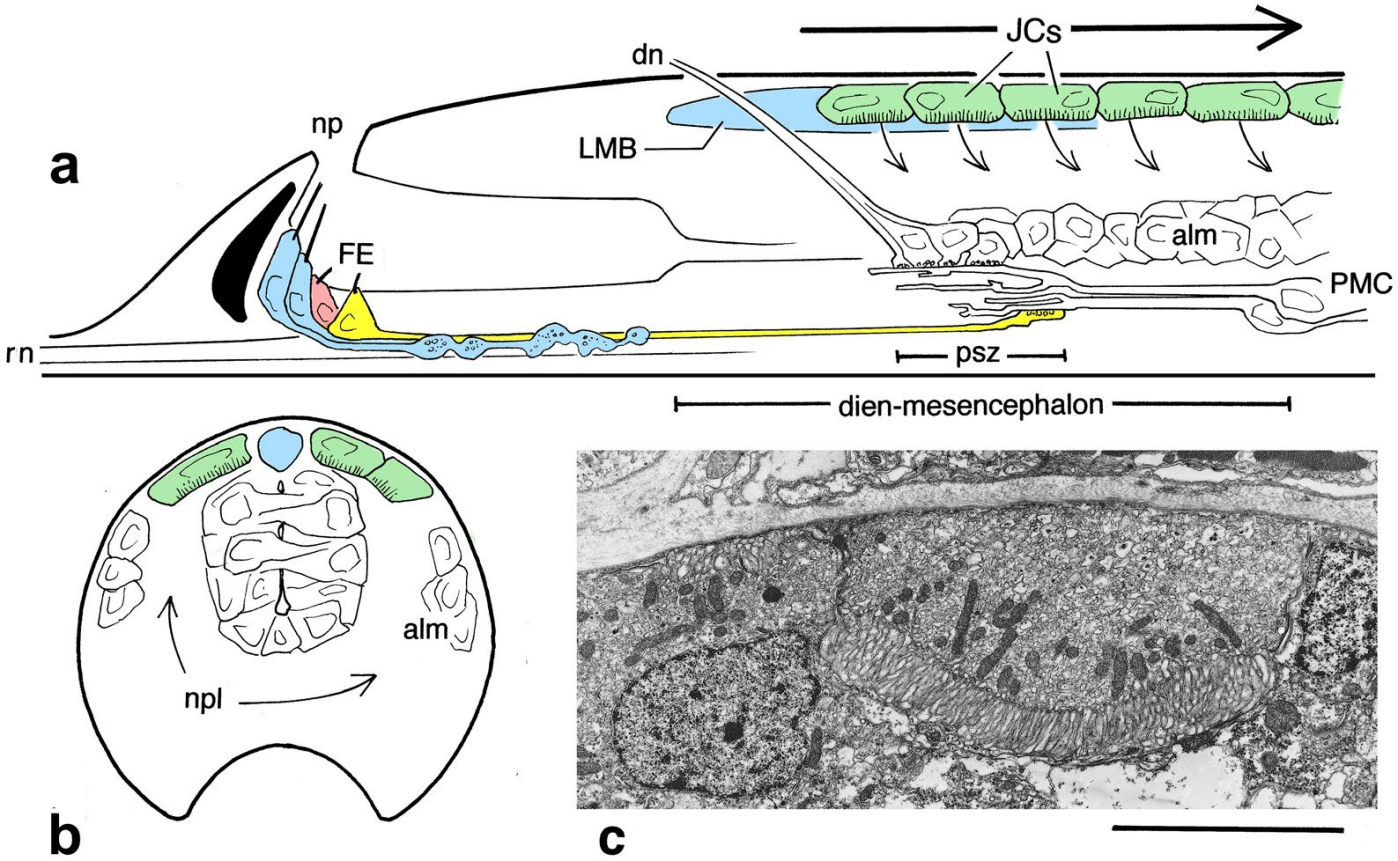
The amphioxus photoreceptors relevant to this account. The photoreceptors of the frontal eye (FE) and lamellar body (LMB), shown in blue, are ciliary, whereas the Joseph cells (JCs, in green) are rhabdomeric. **a** Lateral view of the anterior nerve cord from the frontal eye and neuropore (np) to the end of somite 1, a composite diagram combining features from multiple developmental stages. Data on the frontal eye come from TEM reconstructions of pathways in young *Branchiostoma floridae* larvae, which document a projection from one putative retinal neuron (4L, yellow) to the primary synaptic zone (psz) near the center of the dien-mesencephalon as defined by molecular markers; there 4L synapses to dendrites belonging to the large paired neurons of the primary motor center (PMC). External sensory inputs to the psz are principally from fibers in the rostral nerve (rn), the anterodorsal nerve (dn), and the anterior group of dorsal bipolar neurons, included here as the anterior-most cells of the anterolateral group of migrated neurons (alm), which enlarges as the larvae mature. The Joseph cells are features of older larvae and the adult, and eventually extend forward to the front of the lamellar body according to data from *B. lanceolatum*. The assumption here is that output from the Joseph cells is directed to the cells and neuropile immediately beneath, and is local, so a moving light or passing shadow (large arrow) would be converted into a traveling wave of neural activity in adjacent neurons and neuropile (small arrows). The lamellar body could also play a role here, although, as a putative pineal homolog [10], its involvement in functions related to vision seems less likely, and its output appears to be predominantly to more ventral parts of the neuropile. **b** Cross section of the nerve cord of a juvenile stage at the level of the caudal dien-mesencephalon, showing the position of the lamellar body and Joseph cells, and the much larger zone of neuropile (npl) in the juvenile cord compared with young larvae. **c** TEM image of two adjacent Joseph cells, showing the microvillar array of one. Scale bar = 5 μm

The situation in amphioxus is complicated by the presence of multiple photoreceptor systems ([10], see Fig. 1), among which is a set of rhabdomeric receptors, known as Joseph cells, that form a contiguous layer along the dorsal surface of the nerve cord in the head region of late-stage larvae and the adult. The hypothesis developed here is prompted by the observation that the Joseph cells are positioned in a way that, in principle, would allow them to detect shadows moving across the anterodorsal surface of the body. Gene expression patterns in the developing neural tube show that the location of the anterior-most members of the Joseph cell series corresponds to the roof of the amphioxus homolog of the caudal diencephalon plus the midbrain (the dien-mesencephalon, or DiMes, see [11, 12]), so they occupy a region that, in vertebrates, gives rise to both the optic tectum and pretectum, as well as the thalamic nuclei involved in visual processing. Rhabdomeric receptors are also present in this same region in the brain of salps, a group of pelagic tunicates [13, 14], suggesting the role the dorsal surface of the dien-mesencephalon plays in light reception could be a shared ancestral feature of chordates. It follows from this that a similar set of rhabdomeric photoreceptors, now lost, could have been present in ancestral vertebrates, or at the very least, that there is some feature of the dorsal dien-mesencephalon, predating vertebrates, that predisposes it to become involved in processing visual information. To examine this proposition, I first address the problem of how the amphioxus frontal eye might be restructured to yield something more like a retina. I then consider whether the retinal projection to the dorsal dien-mesencephalon might have been a way for retinal axons to access and co-opt neural circuits that evolved earlier to serve a preexisting dorsal photoreceptor system. In this scenario, the original function of such a system and of the eyes as well would have been for motion detection, with the capacity to form an image being at first a secondary, and perhaps fortuitous consequence.

## The ancestral retina: converting from one dimension to two

The frontal eye of amphioxus has two rows of photoreceptors (blue in Fig. 1a), with an adjacent smaller row of interneurons (red) and a yet smaller row (Row 4, yellow) consisting of two neurons with axons. The photoreceptors project to the rostral nerve and have either local non-synaptic terminals (Row 1, see [7]), or fibers that lack synapses and, as yet, clearly defined targets (Row 2, see [10]). Peripheral fibers in the rostral nerves project further caudally and target the dendrites of PMC neurons, providing a route by which the photoreceptors could potentially influence locomotory control at one remove. The only other output pathway so far documented from the frontal eye involves the neurons in Row 4, one of which (4L, see Figs. 5B and 7 in [15]) has been shown to project to approximately the midpoint of the dien-mesencephalon, where it synapses directly, as shown in Fig. 1a (see Fig. 6D in [15] and Fig. 5B, C in [16]), to dendrites belonging to the PMC neurons responsible for initiating the escape response. This result is based on serial TEM of one specimen, with partial confirming data from a second, so less is known about variability between specimens than one would like. Despite this caveat, the connection between 4L and more caudal targets is well defined and synaptic, which makes it noteworthy, because surrounding neurons mostly lack synaptic contacts, and potentially important from a functional standpoint.

**Fig. 2:**
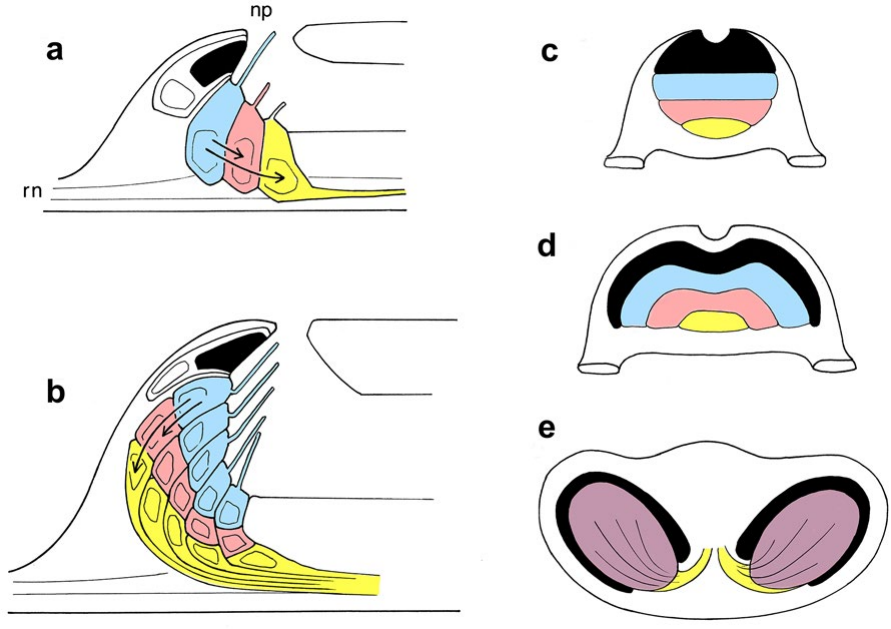
**a**, **b** Steps in the conversion of the frontal eye, with cells arranged in rows, to a two-dimensional receptor array; simplified to show one row each of pigment cells (black), photoreceptors (blue), retinal interneurons (red) and output neurons, i.e., ganglion cell homologs (yellow). **a** The frontal eye in side view, showing the retinal and ganglion cell homologs being produced in sequence (arrows) from a precursor which then terminally differentiates into a photoreceptor. This is a reasonable assumption, given the morphology, but has yet to be proven. All cells contact the ventricular surface in this arrangement, so only one-dimensional receptor arrays are possible. **b** For comparison, a hypothetical, more advanced condition where the precursors have proliferated in two dimensions, i.e., in both the plane of this section and perpendicular to it. Their retinal and output-cell progeny are now produced by basal divisions, and so lack any connection to the ventricular surface of the neural epithelium. This yields a multilayered two-dimensional array where every photoreceptor has direct access to an output pathway. **c**–**e** A hypothetical sequence to show how an amphioxus-type frontal eye (**c**, colors as above) might expand by increasing the length of each transverse row and begin separating into two fields (**d**) to produce, with the organizational changes shown in (**b)**, a flat layered array (shown in purple to indicate a sandwich of blue, red and yellow layers, with a rim of pigment) positioned on either side of the head of a hypothetical early vertebrate (**e**). The frontal eye as shown in (**c**) could itself be reduced from a larger structure in ancestral protochordates, but there is no evidence currently to suggest it was ever multilayered. Abbreviations: neuropore, np; rostral nerve, rn

Because its receptors are organized in transverse rows, the frontal eye is incapable of forming a two-dimensional image. To achieve this, the receptor array would have to be expanded in a second dimension and, to generate a layered structure like the retina, the interneurons have to be displaced from the apical surface of the epithelium (cf. Fig. 2a, b). Assuming that each retinal progenitor cell first produces several classes of interneurons, in fixed sequence, before differentiating terminally as a photoreceptor, the cell lineage need not change. What does change is the division plane, which has to be adjusted so the interneurons are produced basally and remain there. Generating two eyes in place of one is straightforward, as the vertebrate eyes themselves arise from a single, medial primordium. Transitional steps from the current amphioxus condition (Fig. 2c) might have included a laterally expanding bi-lobed receptor array (Fig. 2d), followed by complete separation into two multilayered fields, each with a rim of pigment cells (Fig. 2e).

## Joseph cells and the retinotectal projection

The Joseph cell series begins at the front of somite 2 in larvae but extends forward to the front of the lamellar body in the adult, and hence to the front of the dien-mesencephalon as defined by molecular markers. This places the anterior Joseph cells within the domain from which the optic tectum and pretectum arise, and dorsal to the core decision-making center in ancestral chordates [17, 18]. Very little is known about Joseph cell function beyond the fact that they are indeed photoreceptors [10]. They appear to be primary receptors based on anecdotal reports of axons [19], but have close associations with the axons and cell bodies of adjacent translumenal neurons that could be functionally important. Because they form an extended array over several somites, Joseph cells are ideally positioned to detect changing patterns of light or shadow passing over the anterior part of the body, as would be produced, for example, by the close approach of a predator [10], or if the animal itself moved from deep shadow into light, where it would then be more vulnerable to predator attack. Directing the output from such an array locally, to adjacent cells and neuropile as shown in Fig. 1a, is a logical way to encode information on the time sequence. This is supposition, but is testable, and accords with what is known of projection patterns from the lamellar body in older larvae and juveniles (L. Z. Holland, unpublished data). If true, axons projecting to the roof of the dien-mesencephalon from elsewhere, including from the evolving eyes, would have encountered a zone of neural tissue, effectively a proto-tectum, already organized as a rudimentary spatial map. Co-opting the latter and accommodating to its topology would explain how the retinotectal projection originated and its polarity. A caveat is that one must suppose that the dien-mesencephalic photoreceptors, having been made redundant, were subsequently lost in the vertebrate lineage. My use here of the term “proto-tectum” is not meant to imply that this zone could not also have included the precursor pretectum, which has important visual functions in lamprey [20, 21], and could well predate the tectum as a processing center. From an amphioxus perspective, however, such distinctions are moot, as the Joseph cells appear to be distributed more-or-less uniformly along the whole length of the domain from which both structures arise.

**Fig. 3:**
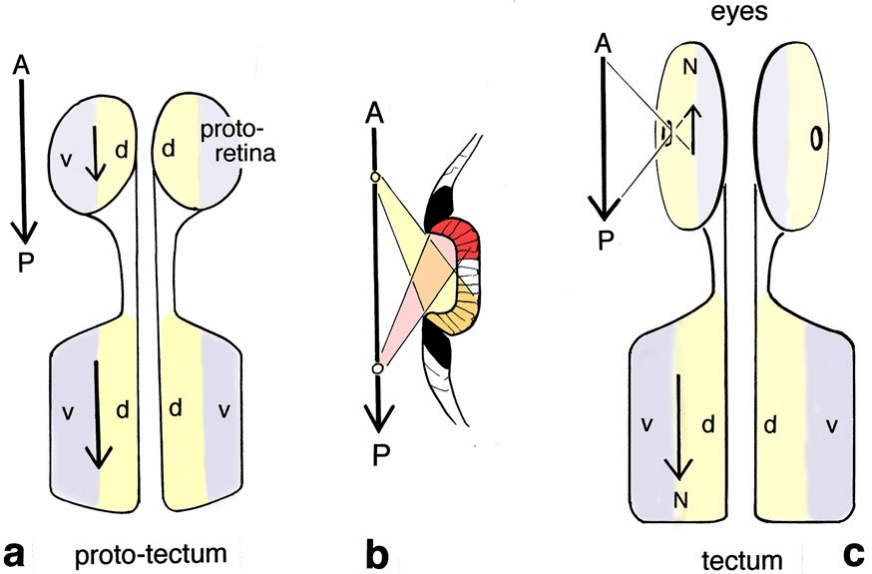
Conservation of tectum topology during the transition from a pair of flat receptor arrays to image-forming eyes. For the sake of argument, the projection is shown as ipsilateral. Contralateral projections predominate in vertebrates, but when and why this feature evolved is beyond the scope of this account. **a** The starting point, a pair of flat receptor fields (each a proto-retina, as in Fig. 2e) with projections to a proto-tectum. The assumption is that the latter already receives input from an overlying array of dorsal photoreceptors, so a shadow moving across the receptor field in an anteroposterior direction (A-P, arrow) or dorsoventrally (d-v, in contrasting colors) would map to the proto-tectum with no change in orientation. Assuming also that fibers from each proto-retina accommodated to this, all arrows would point in the same direction. **b** Image reversal in a flat receptor array with a raised pigmented rim, to show how differential shading by a rim of even modest height reverses the polarity of the response: a light source moving in an anterior-to-posterior direction (yellow to red) stimulates the receptors in a posterior-to-anterior sequence (yellow first, red second). **c** The projection from an image-forming eye whose aperture, with or without a lens, reverses the visual field. Mapping the output to a tectum where the topology is fixed relative to the external world requires the projection to reverse the image a second time, which in fact is what the vertebrate retinotectal projection does [22], so the nasal quadrant of the retina, for example, (*N*, shown as anterior in this diagram) would map to the caudal tectum

The premise here then is, that there was a proto-tectal map predating the arrival of innervation from the eyes, whose topology, reflecting the original rhabdomeric input, directly mapped the external world. Shadows passing in an anteroposterior (A-P) direction would produce a corresponding A-P wave of activity in the proto-tectum, and similarly for shadows moving transversely, i.e., across the dorsoventral (d-v) axis. This is illustrated in Fig. 3a, which shows a hypothetical anterior pair of flat receptor fields whose projection to the proto-tectum preserves both A-P and d-v polarity. In vertebrates, the retinotectal map reverses this relationship [22], while the retinal image is itself reversed when registered by the eyes. This double reversal preserves the directional correspondence between the external world and the tectal map (Fig. 3c), a fact noted in the past [e.g., 23], but without a satisfactory evolutionary explanation. An implication of the present analysis is that the two reversals were probably contingent and correlated with the first appearance of a rudimentary optic cup, since even slightly recessing a receptor field relative to a surrounding pigmented rim will invert the stimulus pattern produced by a moving light source (Fig. 3b). The evident utility of recessing the receptor field in this way can be seen in mollusks, where motion detectors in the form of recessed pit and pinhole eyes are widespread, having evolved independently multiple times [24, 25]. One could also argue, from the above analysis, that the ability of the vertebrate eye to form an image may have been a fortuitous consequence of changes that, in the first instance, were adaptations to improve the detection of motion. This inserts an additional step into the evolutionary sequence generally proposed for eye evolution [e.g., 26], providing at least provisional support for the idea that the role played by ganglion cells in directional selectivity [27, 28] may be a conserved function that has survived from a very early stage in retinal evolution.

## Abbreviations

alm: anterolateral migrated cell group
dn: anterodorsal nerve
FE: frontal eye
JCs: Joseph cells
LMB: lamellar body
np: neuropore
npl: neuropile
PMC: primary motor center
psz: primary synaptic zone
rn: rostral nerve
TEM: transmission electron microscopy
4L: left Row 4 frontal eye neuron
